# A Mobile Health Intervention to Improve Self-Care in Patients With Heart Failure: Pilot Randomized Control Trial

**DOI:** 10.2196/cardio.7848

**Published:** 2017-08-11

**Authors:** Ponrathi Athilingam, Bradlee Jenkins, Marcia Johansson, Miguel Labrador

**Affiliations:** ^1^ College of Nursing University of South Florida Tampa, FL United States; ^2^ Department of Computer Science and Engineering University of South Florida Tampa, FL United States

**Keywords:** heart failure, mobile applications, self-care, quality of life

## Abstract

**Background:**

Heart failure (HF) is a progressive chronic disease affecting 6.5 million Americans and over 15 million individuals globally. Patients with HF are required to engage in complex self-care behaviors. Although the advancements in medicine have enabled people with HF to live longer, they often have poor health-related quality of life and experience severe and frequent symptoms that limit several aspects of their lives. Mobile phone apps have not only created new and interactive ways of communication between patients and health care providers but also provide a platform to enhance adherence to self-care management.

**Objective:**

The aim of this pilot study was to test the feasibility of a newly developed mobile app (HeartMapp) in improving self-care behaviors and quality of life of patients with HF and to calculate effect sizes for sample size calculation for a larger study.

**Methods:**

This was a pilot feasibility randomized controlled trial. Participants were enrolled in the hospital before discharge and followed at home for 30 days. The intervention group used HeartMapp (n=9), whereas the control group (n=9) received HF education. These apps were downloaded onto their mobile phones for daily use.

**Results:**

A total of 72% (13/18) participants completed the study; the mean age of the participants was 53 (SD 4.02) years, 56% (10/18) were females, 61% (11/18) lived alone, 33% (6/18) were African Americans, and 61% (11/18) used mobile phone to get health information. The mean engagement with HeartMapp was 78%. Results were promising with a trend that participants in the HeartMapp group had a significant mean score change on self-care management (8.7 vs 2.3; t3.38=11, *P*=.01), self-care confidence (6.7 vs 1.8; t2.53=11, *P*=.28), and HF knowledge (3 vs −0.66; t2.37=11, *P*=.04. Depression improved among both groups, more so in the control group (−1.14 vs −5.17; t1.97=11, *P*=.07). Quality of life declined among both groups, more so in the control group (2.14 vs 9.0; t−1.43=11, *P*=.18).

**Conclusions:**

The trends demonstrated in this pilot feasibility study warrant further exploration on the use of HeartMapp to improve HF outcomes.

**Trial Registration:**

Pilot study, no funding from National agencies, hence not registered.

## Introduction

Heart failure (HF) is a progressive disease affecting 6.5 million Americans, with costs exceeding US $39.2 billion annually [1]. Presently, treatments for HF largely comprise drug therapies targeting pathophysiology and complex educational interventions targeting self-care practices [2]. Self-care regimens for patients with HF are complex and multifaceted, and patients often find it hard to understand how to monitor HF symptoms and understand weight fluctuations and to seek care without delay [2,3]. It appears that increased knowledge of self-care alone does not often translate to changes in self-care practice [4]. Poor self-care is associated with delay in seeking care for HF symptoms [5], poor medication adherence [6,7], reduced quality of life [8], increased hospital readmissions, and mortality [7]. A meta-analysis suggested that telemonitoring for HF patients can reduce hospitalization [9]. However, a large Telemedical Interventional Monitoring in HF study demonstrated no significant improvement in HF-related outcomes [10]. Similarly, the large Better Effectiveness After Transition-Heart Failure study that provided remote monitoring intervention with nurse call failed to demonstrate significant reduction in readmission rates [11]. A qualitative study that interviewed 18 patients with HF and 5 health care professionals reported that the remote home monitoring systems for HF have not been widely adopted by patients because the currently available home monitoring devices are not personalized to meet the patients’ needs [12]. In the same study, HF nurses reported that telemonitoring devices offered false hope to patients of being monitored continuously by providers, and the patients lacked development of independent self-care behavior [12]. A recent meta-synthesis of 9 systematic reviews of telemonitoring of physiological parameters and telephone support significantly reduced HF-related readmissions, whereas no impact was reported from telephone-only interventions and a variable effect reported on hospital admissions [13]. These findings resulted in an approach to develop a personalized mobile health (mHealth) coach (HeartMapp) that is easy for use by older adults with HF with physiological monitoring using a chest-worn Bluetooth device.

## Methods

### Conceptual Framework

**Figure 1:**
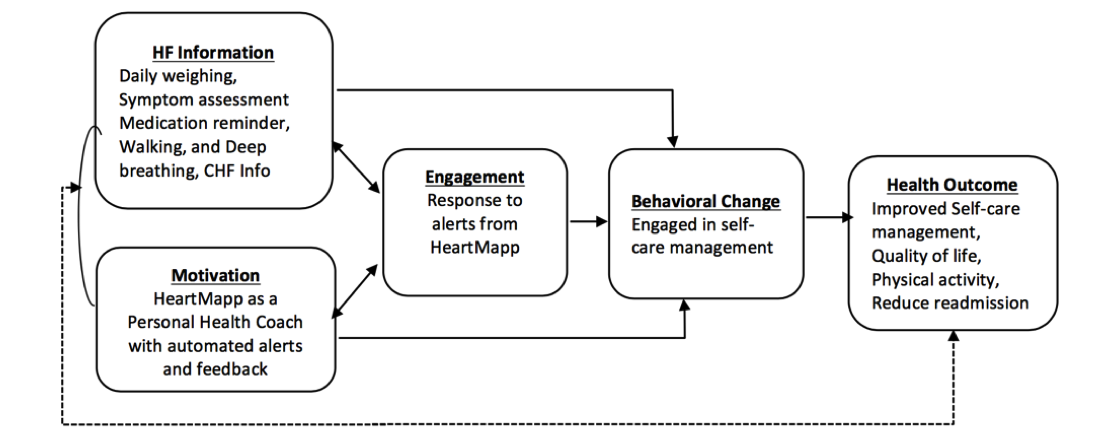
Conceptual framework of information, motivation, and behavior, and patient engagement.

Patients who are engaged as decision makers in their care tend to be healthier and have better outcomes [14]. Thus, in developing a conceptual framework for this research, Carmen’s multidimensional framework of patient engagement was used to achieve persistent behavior change in patients with HF [15]. Patient engagement was augmented with the use of information-motivation-behavioral skills (IMB) model in the development of the conceptual framework [16]. Essentially, the IMB model asserts that people who are well informed and motivated are likely to engage in activities that enhance knowledge and skills needed to perform focused behavior, which allows them to reap greater health benefits [16]. Enabling mHealth has been supported as a pathway to inform patients. This pilot study paired information (HF self-management skills and knowledge) and motivation (using HeartMapp as a health care coach) to promote and support intrinsic motivation in patients and to increase engagement in behaviors required to manage their condition effectively [16]. It is proposed that increased engagement with HeartMapp may reduce symptom burden in patients with HF (see Figure 1).

An iterative patient-centered approach was adopted during the design of HeartMapp by leveraging mobile phones to target individualized alerts focused on patient needs to improve self-care and medication adherence, which could be used in a real-life setting as and when needed. HeartMapp also used a Zephyr BioHamess-3 chest strap [17] that connects to the Android device via Bluetooth to monitor physiological data, including heart rate, heart rate variability, and accelerometer data. During the development process, multiple iterations were made based on feedback from patients with alpha and beta testing and a usability study [18]. On the basis of feedback from patients with HF and providers who cared for them, additional features were added to the HeartMapp system. The HeartMapp mobile system was pilot-tested for feasibility with regard to use by patients with HF.

### Design and Method

This was a pilot randomized controlled trial (RCT). The overall goal of the pilot RCT was to examine feasibility of HeartMapp use by patients with HF and to compute effect sizes for sample size estimation for a larger RCT. The pilot study enrolled 18 participants with HF; the intervention group (n=9) received all 6 features of the HeartMapp, and the active wait-listed control group (n=9) received only HF education (congestive heart failure [CHF] info feature of the HeartMapp). The apps were downloaded onto their mobile phone for daily use. The active wait-listed control group was given access to 4 additional features of the HeartMapp at the 30-day follow-up. Participants were block randomized as (3:3; 2:2; 3:3; 2:2; 3:3; 2:2; 3:3; 2:2).

This is a pilot feasibility trial which received no NIH or other organizational funding; hence, it was not registered.

#### Patient Recruitment

Upon obtaining approval by the University’s institutional review board (IRB), participants were recruited from a tertiary hospital. The IRB-approved brochures were made available at inpatient units. The discharge care coordinators referred potential HF participants for the study.

##### Inclusion and Exclusion Criteria

Textbox 1 below summarizes the inclusion and exclusion criteria. To assure that the eligible participants could properly understand how to use the HeartMapp, all participants were screened for adequate hearing acuity (thresholds of 4000 dB HL [decibels Hearing Level] or better) in the midfrequency range in at least one ear measured using a handheld combination otoscope and audiometer (Audioscope by Welch Allyn) [19]; vision (corrected near visual acuity of 20/50 or better) on the Snellen chart [20]; and assessed for cognitive function using the Montreal Cognitive Assessment (MoCA) [21]. The MoCA is a valid and reliable cognitive screening tool (Cronbach alpha=.83) and has been validated in HF patients in a prior study [22]. Patients who did not own an Android mobile phone were loaned one with Wi-Fi capability.

##### Study Procedures

A research assistant (RA), a research nurse, approached participants who had indicated interest and were ready for discharge. The RA explained the study requirements and sought consent from the participants using the IRB-approved informed consent document. Consented participants were screened for eligibility. Participants who failed screening were referred for further clinical evaluation. Within 3 to 7 days after discharge, 2 RAs scheduled a time to meet the patients in their home. During the home visit, the RAs confirmed their willingness to participate in the study and collected baseline data.

##### HeartMapp Intervention Group (n=9)

**Figure 2:**
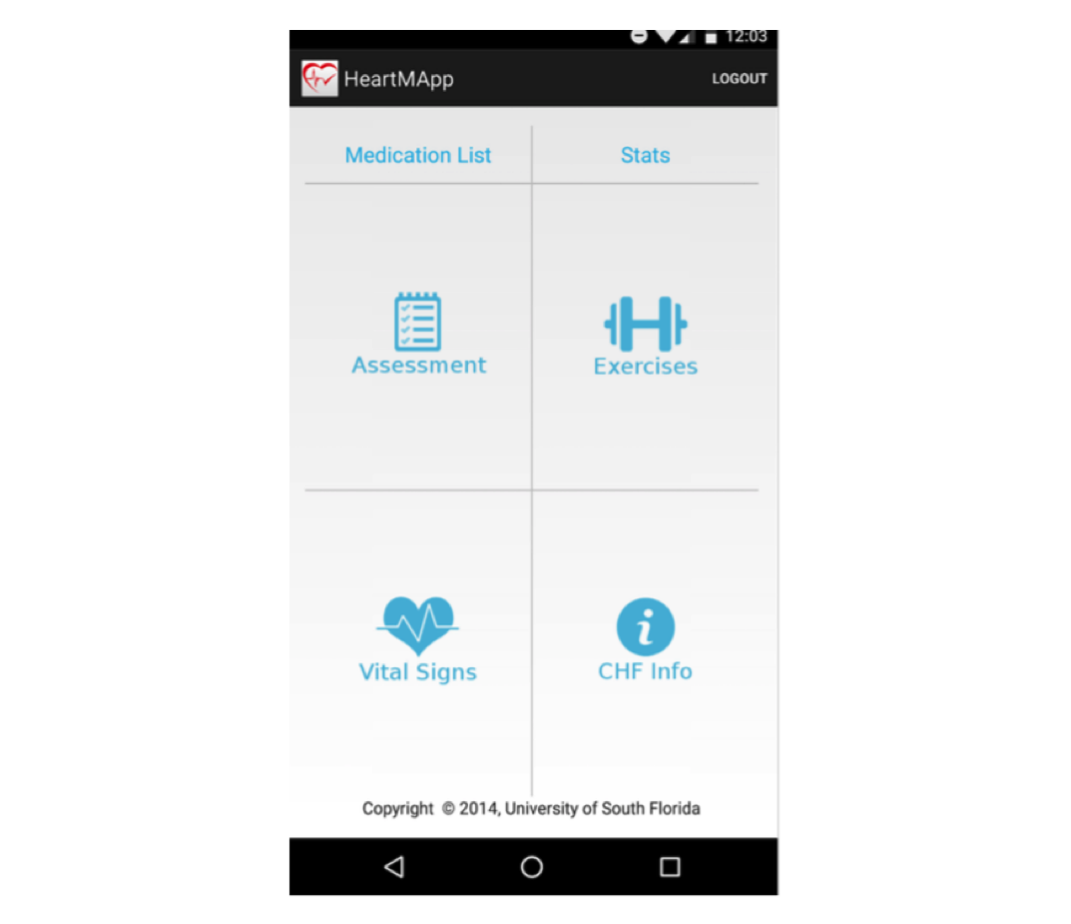
HeartMapp features.

The intervention group had HeartMapp downloaded onto their Android phone or a loaner phone. The participants were trained on HeartMapp features, including daily weighing, symptom assessment, responding to tailored alerts, vital sign monitoring using BioHarness-3 chest strap, HF education (CHF info), and performing breathing exercise and walking. Participants were asked to use HeartMapp daily from home for a total of 4 weeks. The details of the 6 features of the HeartMapp are provided below (see Figure 2).

**Inclusion and exclusion criteria.**

Inclusion criteria

- Clinical diagnosis of congestive heart failure (CHF) as defined by the International Classification of Diseases (ICD-10 codes) and recent hospitalization for CHF
- New York Heart Association (NYHA) classification II-III
- Adults aged 30 years or above
- Ability to speak, understand, and read English
- Vision must be 20/50 or better
- Adequate hearing
  - Hearing thresholds of 4000 dB HL or better
- Must be willing to use the mobile app (HeartMapp or CHF info) and the Bio-Harness-3 chest-worn Bluetooth device

Exclusion criteria

- Listed for heart transplant as status 1A; or on home milrinone or dobutamine infusion
- Enrolled in a palliative or hospice care program
- History of stroke within the past year
- Major disability such as aphasia
- Uncontrolled psychiatric disorders
- Cognition: Montreal Cognitive Assessment score <20
- Living in a setting where they are not able to independently engage in self-care (eg, skilled nursing facility)

###### Medication Tracker

The medication tracker allows patients to add and edit their medications and activate reminders to take medications (eg, push notification). This feature can also assist home health nurses to update their patients’ medications list collaboratively. Given the success of related product features in other populations (eg, Text4Baby), this mechanism may improve behavioral outcomes in HF [23]. In fact, a meta-analysis of 16 studies reported that text messaging significantly improved medication adherence (odds ratio 2.11; 95% CI 1.52-2.93; *P*<.001) among patients with chronic diseases [24].

###### Assessment of HF Symptoms

HeartMapp provides a self-care management point-of-care tool where tailored automated reminders are sent to patients to check their weight and complete HF symptom assessment questions. Although symptoms are the hallmarks of severity of HF, patients’ experience of symptom clusters and symptom intensities vary and may reflect the personal and social experiences of illness, cultural differences in the interpretation of symptoms, and responses to the illness—all of which may influence HF self-care practices and care-seeking behavior [25]. Evidence suggests that symptom recognition may be impaired in the elderly population [26,27]. Furthermore, patients often misinterpret symptoms of HF and decline to seek early medical care, which results in higher readmission rates.

Once users have registered with HeartMapp with baseline height, weight, and health care provider information, they receive tailored daily prompts and are provided access to the assessment window to check weight, blood pressure, and answer the short questionnaires on HF symptoms. HeartMapp then classifies users via the HF severity index based on the New York Heart Association (NYHA) functional classification [28]. The algorithm in HeartMapp uses the information entered by patients on weight and HF symptoms to classify the patients into (1) Green Zone, if HF symptoms are reported as stable or no change in weight; (2) Yellow Zone, if HF symptoms are reported as mild and a weight gain of 3 pounds in 1 day or 5 pounds in a week; (3) Orange Zone, if symptoms are moderate and a weight gain of more than 5 pounds; or (4) the Red Zone, if HF symptoms require immediate attention. On the basis of the information gathered, the HeartMapp provides an alert with the following feedback at the end: the Green Zone (“stable with no change-continue current self-care practices”); the Yellow Zone (“take an extra dose of water pill if prescribed by your doctor and call the home care staff if receiving home care or your doctor’s office”); the Orange Zone (“call home care staff or the doctor’s office now” and open the phone number on the file provided during registration of the app to alert the study coordinator); and the Red Zone (automatically dials 911 and sends a panic alert to the study coordinator and home care staff). Random text alerts are designed based on key evidence from HF literature and patient preference. In fact, a pre-post pilot study that provided text messages to patients with HF (n=15) reported an increase in mean composite score of HF self-care maintenance from 49 to 78 (*P*=.003), and self-care management increased from 57 to 86 (*P*=.002) at 4 weeks [29]. Furthermore, regular app usage provides useful metrics of patient engagement.

Patients with HF have an increased prevalence of cognitive impairment with 4 times higher risk of developing dementia compared with healthy adults of the same age. The etiology is believed to be of vascular origin because of hypoperfusion to the brain resulting from reduced cardiac function. Therefore, HeartMapp includes a memory screener and simple naming tasks to measure cognition using Boston Naming Test criteria with 9-items [30]. Patients with early vascular dementia caused by cerebral hypoperfusion in HF displayed more naming errors overall [31].

###### Physiological Exercises

The exercise feature includes animated biofeedback deep breathing exercises and walking. Deep breathing interventions deployed in HeartMapp use biofeedback mechanism to reset the autonomic nervous system by reducing sympathetic activity and increasing parasympathetic activity [32,33]. Also, HF patients have 2 to 3 times higher incidence of depression compared with general population [34]. Therefore, HeartMapp is designed to teach patients about using biofeedback to attain 6 breaths per min and offer feedback on their performance. Controlled breathing at 6 breaths per min, compared with spontaneous breathing at 15 breaths per min, has been shown to reduce fluctuations in blood pressure and significantly increase baroreflex sensitivity measured by spectral analysis electrocardiogram in a study among 81 patients with HF (from 5.0 [SD 0.3] to 6.1 [SD 0.5] ms/mm Hg, *P*<.001), compared with 21 healthy controls [35].

HeartMapp encourages walking 3 to 4 times a week and provides feedback to patients on performance. The walking test is a simple measure of functional capacity that predicts survival in patients with moderate HF. Patients with HF who have an ejection fraction of less than 30% have been shown to improve exercise tolerance and physical ability [36]. Therefore, HeartMapp is built to encourage physical activity and tracks distance walked. HeartMapp also gives feedback to patients on their performance by utilizing the distance walked by individuals based on their age, gender, height, and weight.

###### Performance Tracker (Stats)

Performance tracking features in the HeartMapp include a graphical module that displays trends in patient performance, including weight, blood pressure, HF symptoms, and physiological measures such as heart rate and exercise activity. These data serve as a tool for triage by home health nurses to understand early decline and thus prevent readmission and or emergency room visits by quickly intervening (eg, by providing an extra dose of diuretic or an early office visit for evaluation).

###### Vital Signs Monitoring

Monitoring of vital signs is captured via integration of an open application programming interface (API) from BioHarness-3 from Zephyr technology [17]. Because wearable devices for continuous vital sign monitoring are expected to revolutionize health care services, particularly in the home setting [37], the BioHarness-3 was used to obtain heart rate and accelerometer data.

###### HF Education (CHF Info)

HF education (CHF-info feature) includes 10 educational modules specific for HF and common chronic diseases associated with HF. Evidence indicates that traditional patient education using printed materials does not support self-care skill development; thus, novel patient-teaching strategies and persistent engagement of patients are needed to support the development of tactical and situational skills [38]. The HF-info feature in the HeartMapp includes audio-enabled interactive teaching tools on the nature of heart failure, importance of low-salt diet, exercise regimen, HF medications, and managing other chronic diseases or conditions and feelings about HF as well as heart and brain connection. Theory-based development and beta testing of embedding HF education within HeartMapp has been published [39].

##### Active Wait-Listed Control Group (n=9)

**Figure 3:**
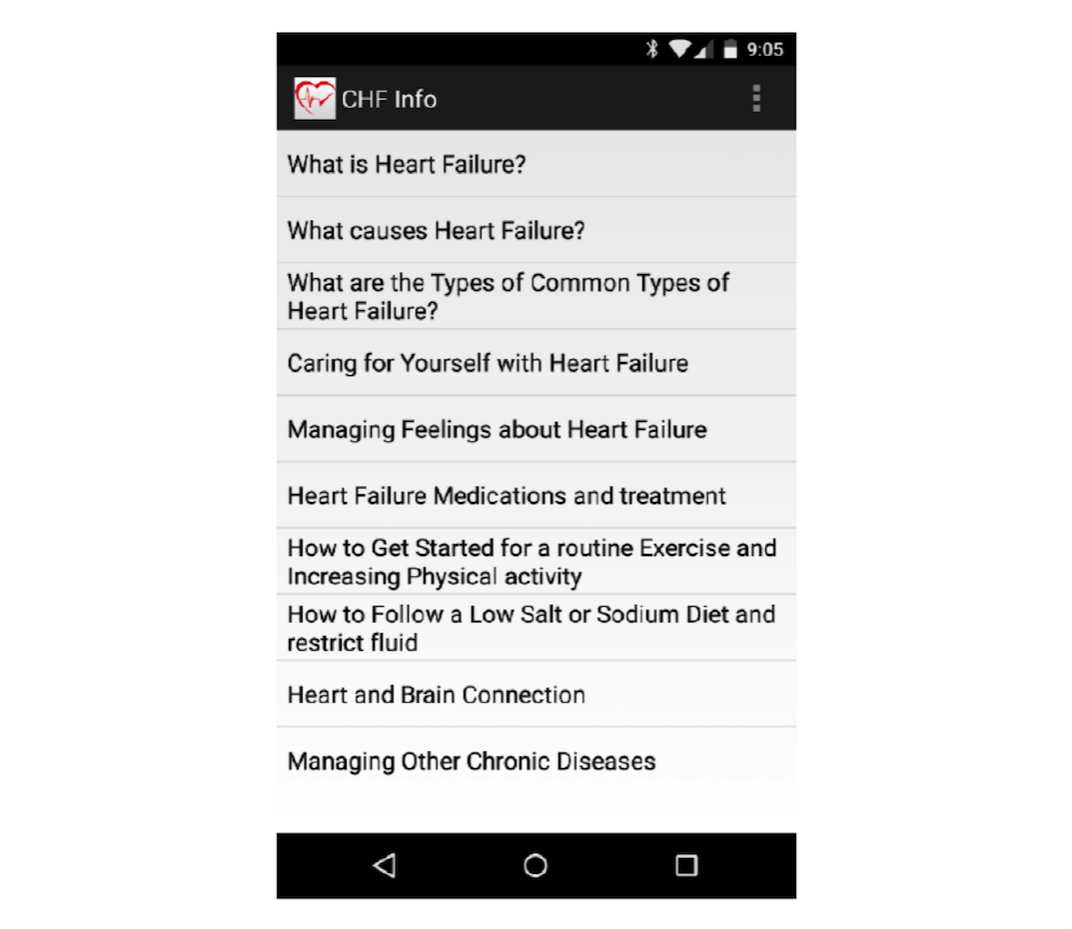
Congestive heart failure (CHF) info app.

The control group (n=9) received only HF info downloaded onto their mobile phone, as shown in Figure 3. These patients were encouraged to use 3 modules per week and complete all 10 modules by 4 weeks. Participants were assured that they will receive the additional features at the 4-week follow-up. At the end of the follow-up, they were given access to 4 features of the HeartMapp, with the exception of vital signs monitoring using the chest strap BioHarness-3.

### Patient Safety and Monitoring

A study coordinator (doctoral student and nurse practitioner) called all participants 3 times a week during the first week and once a week for the remaining 3 weeks to make sure that they completed their tasks and answered any questions that came up, checked the dashboard daily to monitor participants’ progress, and triaged for further management per protocol. No participants during the study period were referred to a cardiologist (project consultant) for HF symptoms warranting immediate attention.

### Outcome Measures

*Patient engagement* and HeartMapp usage were assessed based on the duration for which the participants accessed HeartMapp features that were timestamped and recorded on a secured website.

*Self-confidence in using HeartMapp* questionnaire, which was designed based on Bandura’s self-efficacy scale [40], was completed by participants who used HeartMapp at the 30-day follow-up. This questionnaire has 10 Likert scale questions and was validated in a prior usability study (*r*=.98) [18].

The *usability of HeartMapp* questionnaire validated in a prior study (*r*=.468-.635) [18] has a total of 25 questions in a 5-point Likert scale [16]. This questionnaire assessed the ease of use, problem-solving capabilities, accuracy and clarity of presentation, and satisfaction with the use and design of HeartMapp. Open-ended questions were included at the end for comments on specific task needs.

Self-care behavior in HF was assessed using the *Self-Care of Heart Failure Index* [41] that has been used in multiple studies involving HF patients. Reliability of the Self-Care Maintenance subscale had a score of (*r*=.56), whereas the scores of Self-Care Management and Self-Care Self-Confidence were (*r*=.70) and (*r*=.82), respectively [41]. Higher score indicates better self-care.

Medication adherence was assessed using the 8-item self- *administered Morisky Medication Adherence Questionnaire*, with lower score indicating better adherence (*r*=.83) [42].

The HF-specific knowledge was assessed using the *Atlanta Heart Failure Knowledge* Test [43]. This questionnaire has 30 questions with a possible score of 0 to 30 (*r*=.84 for patients); higher score indicates better knowledge [43].

Participants’ perception about clinical changes in quality of life was measured using the *Kansas City Cardiomyopathy Questionnaire* (KCCQ) [44]. This 23-item scale has 5 clinically relevant domains for persons with HF, which include physical limitations, symptoms (frequency, severity, and change over time), quality of life, social interference, and self-efficacy (*r*=.66-.95) [44]. Lower score indicates worse symptoms and worse quality of life.

*Patient Health Questionnaire (PHQ-9),* a self-administered depression scale, which scores each of the 9 Diagnostic and Statistical Manual of Mental Disorders, Fourth Edition criteria as 0 (not at all) to 3 (nearly every day), was used to examine depression [45]. Higher score indicates worse depression. Scores of 5, 10, 15, and 20 represent cutpoints for mild, moderate, moderately severe, and severe depression, respectively. A PHQ-9 score of ≥10 has a sensitivity of 88% and a specificity of 88% for major depression and has been validated in 247 patients with HF (*r*=.82) [45].

Demographic variables including age, gender, ethnicity, and marital status as well as clinical variables on medications, ejection fraction, and NYHA were collected at baseline [46,47]. Illness burden was measured using *Cumulative Illness Rating Scale* that measures illness burden on a 5-point scale with scores ranging from 0 to 56, which demonstrated an interclass correlation coefficient of .78 on illness burden [48]. The Duke-UNC short-version 8-item *Functional Social Support Questionnaire* (FSSQ) assessed social support (*r*=.50-.85). The FSSQ uses a 5-point Likert scale (1=much less than I would like and 5=as much as I would like) [49].

### Data Analysis

Baseline and 30-day follow-up data were collected at participants’ home. Partial eta squared for effect sizes were computed from group mean differences using linear regression. Furthermore, *t*-tests were performed to compare mean differences between intervention and control groups and between time points (post- and preintervention scores). Exploratory correlation analyses were performed to evaluate changes in outcome measures relative to baseline performance on assessments.

## Results

Of the 20 patients who were screened for eligibility, 1 participant gave us a wrong home address and could not be tracked, and another participant refused to be a part of the study after eligibility screening. All participants met the inclusion criteria, including normal hearing and vision as well as normal cognition with mean MoCA score of 26.3 (SD 2.4). A total of 18 participants met the inclusion criteria and were block randomized to HeartMapp (n=9) and active wait-listed control (n=9) to HF education (CHF info, one feature of the HeartMapp) on the mobile phone for the first 30 days. Only 13 participants (72%), 7 in the HeartMapp group and 6 in the HF info group completed the 30-day follow-up. As mentioned earlier, an Android phone was loaned for the study period for the participants who did not own a phone.

### Demographic Data

Participants’ mean age was 53.06 years; 56% (10/18) of the participants were females, 61% (11/18) lived alone (divorced, never married, or widowed), 33% (6/18) were African Americans, and 17% (3/18) were Hispanics. Only one participant, 6% had less than high school education, 89% (16/18) had non-ischemic cardiomyopathy, and 67% (12/18) had HF for over a year. Mean ejection fraction was 28%, 67% (12/18) were in HF stage C, and 61% (11/18) were in NYHA class II (see Table 1 for detail).

On the illness burden scale measured by the Modified Cumulative Illness Rating Scale, 78% (14/18) of participants rated cardiac condition as severe to extremely severe (3-4) burdensome; whereas 17% (3/18) reported severe burdensome for endocrine problems (diabetes), 11% (2/18) rated severe burdensome for psychiatric problems and kidney diseases. All participants owned a mobile phone, one participant owned an Apple phone, and 63% (11/18) used a mobile phone to get health information.

### Mean Difference in HF Outcomes Between Intervention and Control Groups (Post-Pre Scores) and Baseline Versus 30-Day Follow-Up Scores

Mean for baseline and 30-day follow-up scores were computed, which demonstrated an improvement in almost all outcomes. An independent *t*-test was used to compute mean change scores of HeartMapp and control group at baseline and 30-day follow-up (30-day score-baseline score) for the 72% (13/18)) participants (7 HeartMapp and 6 control group) who completed the 30-day follow-up. The results showed statistical significance in few of the outcomes.

In general, trend on improvements were noted at 30-day follow-up on all outcomes among groups. Among patients in HeartMapp group, a mean score change on self-care management by 8 points was noted, whereas the control group improved by 2 points (*P*=.01); self-care confidence improved by 7 points in HeartMapp group compared with an increase of 2 points among controls (*P*=.03). Mean score change on HF knowledge among HeartMapp group was 3 points, whereas the control group declined (*P*=.04). Quality of life measured by KCCQ declined by 2 points in HeartMapp group, whereas the control group declined by 9 points (*P*=.18). Medication adherence improved among both groups (see Table 2).

### Effect Size Calculation Using Partial Eta Squared

We computed the partial eta squared as the effect sizes from group mean differences using linear regression. The partial eta squared indicates the percentage of variance in each of the effects (or interaction) and its associated error that is accounted for by that effect (or interaction) [50]. The partial eta squared indicated small to moderate effect sizes (self-care 0.249; HF knowledge 0.337; quality of life 0.156; depression 0.262; and medication adherence 0.036).

### Patient Satisfaction and Usability of HeartMapp

Seven participants from HeartMapp group who completed the 30-day follow-up were asked to rate the HeartMapp usability questionnaire that assessed HeartMapp features and self-confidence in using HeartMapp questionnaire (see Table 3).

### Engagement With HeartMapp

The details on participants’ access to HeartMapp features were stored and time stamped on a secure website for analysis. A mean of 78% engagement with HeartMapp was noted among all participants randomized to HeartMapp. Of the nine participants randomized to HeartMapp, 43% (4/9) accessed HeartMapp daily and completed the assessment of HF symptoms and exercise (walking) daily. Five participants accessed HeartMapp features over 24 days or 80% of the time. The multivariate regression analysis did not predict any association with patient engagement or HeartMapp access time with HF outcome. However, other features of the HeartMapp were accessed by participants inconsistently. The features consistently accessed were the medication tracker and breathing exercise. The least accessed feature was the vital signs monitoring using the chest-worn Bluetooth device (BioHarness-3).

**Table 1:** Sample characteristics.

Characteristics		n (%) (N=18)	Mean (standard deviation)
**Age, in years**			
	≥65	12 (66.7)	53.06 (4.02)
**Gender**			
	Female	10 (55.6)	
**Living status**			
	Never married	4 (22.2)	
	Married	7 (38.9)	
	Widowed, separated	7 (38.9)	
**Race**			
	White	9 (50)	
	African American	6 (33.3)	
	Hispanic	3 (17)	
**Education**			
	More than 1 or more college	11 (61.1)	
	High school	6 (33.3)	
	Less than high school	1 (5.6)	
Ejection fraction			28.09 (14.61)
**Etiology of HF^a^**			
	Nonischemic	16 (88.9)	
**NYHA^b^** **class**			
	Class I	1 (5.6)	
	Class II	11 (61.1)	
	Class III	6 (33.3)	
**HF stage**			
	Stage B	6 (33.3)	
	Stage C	12 (66.7)	
Body mass index			36.55 (13.12)
Cognitive score at baseline (MoCA^c^ score)			26.33 (2.40)
Social support at baseline (FSSQ^d^ score)			34.94 (6.63)
Illness burden (MCIR^e^ score)			22.72 (4.16)
Owned a mobile phone		18 (100)	
**Type of phone**			
	Apple iPhone	1 (5.6)	
	Android	11 (61.1)	
	Analog	6 (33.3)	
**Use of mobile phones**			
	Very well	7 (38.9)	
	Moderately well	11 (61.1)	
**Mobile phone use**			
	Texting	18 (100)	
	Health information	11 (61.1)	

^a^HF: heart failure.

^b^NYHA: New York Heart Association.

^c^MoCA: Montreal Cognitive Assessment.

^d^FSSQ: Functional Social Support Questionnaire.

^e^MCIR: Modified Cumulative Illness Rating.

**Table 2:** Mean difference between HeartMapp and control groups at baseline and 30 days.

Outcome measures	HeartMapp	HeartMapp	Control	Control	*t*	Significant *P* value
	Baseline	30-days	Baseline	30-days		
	Mean (SD)	Mean (SD)	Mean (SD)	Mean (SD)		
Self-Care Maintenance	22.86 (2.41)	28.29 (2.81)	17.67 (3.61)	22.83 (5.56)	0.083	.93
Self-care Management	12.00 (2.77)	20.71 (1.11)	9.67 (2.66)	12.33 (3.03)	3.38	.01
Self-care Confidence	12.43 (2.94)	19.46 (4.05)	14.67 (3.01)	16.50 (2.07)	2.53	.03
Med. Adherence	3.00 (1.73)	3.23 (1.64)	3.50 (1.65)	2.33 (1.50)	0.408	.53
HF Knowledge	24.29 (1.56)	27.28 (2.29)	24.83 (2.13)	24.17 (1.71)	2.37	.04
Quality of Life	20.71 (9.09)	23.43 (6.50)	19.69 (7.64)	27.5 (8.52)	1.43	.18
Depression	8.14 (5.60)	7.00 (6.13)	8.50 (5.36)	3.33 (2.94)	1.97	.07

**Table 3:** Patient Self-Confidence and Usability of HeartMapp.

Descriptors for measures	Mean (SD)
Ease of using the HeartMapp	24.86 (4.02)
Accuracy of contents of HeartMapp	19.43 (4.50)
Use and design of the HeartMapp	17.86 (2.67)
Problem-solving feature of HeartMapp	15.57 (6.24)
Self-confidence in using the HeartMapp	18.00 (15.74)

### Open-Ended Questions on the Usability Scale

Assessment of open-ended questions using descriptive and thematic analysis suggested that 86% (8/9) of the participants preferred a wrist-worn Bluetooth device. The chest-worn Bluetooth device (BioHarness-3) was reported by more than half of the participants (>50%) as uncomfortable, and all the participants reported poor battery life, with the need to charge the device twice a day. This indicated noncompliance in using the chest strap.

## Discussion

### Principal Findings

The result of this pilot study is promising in that the HeartMapp intervention showed trends in improving several HF outcomes, especially self-care management, self-care confidence, and HF knowledge. A comprehensive review of 34 commercially available mobile apps for HF symptom monitoring and self-care rated Heart Failure Health Storylines, Symple, ContinuousCare Health App, WebMD, and AskMD as the highest performing apps and recommended future studies to test the usability and effectiveness of the available apps [51]. On the other hand, several intervention studies demonstrated inconclusive results, including the Heart Smart symptom training [52], the large Comparison of Outcomes and Access to Care for Heart Failure study (n=1023) on intense disease management [53], motivational interviewing in HF [54], transitional care [55], and a nurse-led cognitive behavioral intervention [56] that demonstrated no difference or improvement in HF outcomes. Also, the Alere Day-Link monitor that used electronic scale and a computer-based individualized symptom response [57] also did not demonstrate significant improvement in HF outcomes. Thus, a large-scale study is warranted to test HeartMapp for functionality and improved HF outcomes.

A systematic review on remote telemonitoring interventions reduced the relative risk of all-cause mortality (0.60-0.85) and HF-related hospitalizations (0.64-0.86) compared with usual care. Improvements in HF-related hospitalizations appeared to be more pronounced in patients with stable HF (hazard ratio 0.70; 95% CI 0.34-1.5), indicating appropriate selection of patients who are not in the refractory or end-stage HF [58]. This is possibly true in this pilot study, as 61% of the patients were in NYHA class II.

A pilot study that offered text messaging to HF patients after discharge demonstrated significant improvement in HF self-management [29], as seen among participants who used HeartMapp. Similarly, Weight and Activity with Blood Pressure Monitoring System (WANDA), which comprises wireless sensors and a mobile device, enabled the patients to reduce 5.6% of weight and blood pressure values that were out of the acceptable range; however, this system is still under updates before becoming commercially available [59]. Therefore, the trends observed on improved HF outcomes in this pilot study warrant an exploration in a well-designed RCT.

The result of this pilot study indicated that only 72% of participants completed the 30-day follow-up, which is very low and the reason most reported was the use of BioHarness-3 chest strap. An attrition rate of 35% has been reported in prior studies among HF population [60]. Those who completed the follow-up did not use the BioHarness-3 consistently. A mean of 78% engagement with HeartMapp was noted among all participants randomized to HeartMapp. Again, this may be because of the challenges faced in using the BioHarness-3 chest strap. The engagement with HeartMapp observed in this sample is comparative with the national data from Common Wealth Fund (2016) that showed engagement at 16% for Android apps and 6% for iPhone operating system (iOS) and usefulness at 43% for iOS apps and 27% for Android apps [61]. Although these are general data, the 78% engagement with HeartMapp seen in this small sample could be because of the features tailored specifically for HF patients.

One in 6 (15%) consumers in the United States currently uses wearable technology, mostly smart watches or fitness bands [37]. Over 80% of the study participants reported a preference for wrist-worn Bluetooth device to track vital signs, physical activity, and sleep, but they did not use the chest-worn Bluetooth device. Therefore, the team is currently testing several wrist-worn Bluetooth devices with open API in the laboratory (ie, Microsoft Band-2, Fitbit Charge HR, and Moto 360). An open API allows all developers by offering one piece of software to interact with another piece of software. Thus, the research team hopes to tap into the consumer health wearables using one of the wrist-worn devices for a larger clinical trial to test with HeartMapp.

This pilot study only enrolled a small sample of 18 HF patients, with only 72% completing the 30-day follow-up; thus, the results are not generalizable. The small number of participants rated high satisfaction with the ease of using HeartMapp, which supported the results of the prior usability study [18]. Gerber and colleagues [62] supported that older adults and marginalized segments of the population have better access and uptake of mobile technology. However, technology use has been observed to decrease significantly with greater limitations in physical capacity such as vision impairment and memory limitations [63]. Therefore, the inclusion screening criteria was stringent in patients for vision, hearing, and cognitive function, which needs further exploration in a larger study. Lessons learned in this pilot study helped the team to refine a few features in HeartMapp and to overcome shortcomings in a future larger trial.

### Conclusions

There is currently an urgent unmet need for HF-targeted patient-centered interventions that are easy to use by older adults with HF who experience cognitive difficulties and lack social support. Participants in this study reported that HeartMapp is easy to use. However, there is need for updates on integrating a wrist-worn Bluetooth device for monitoring of vital signs, which the team is currently addressing. In the current market, there are a few other apps being tested that track patients’ self-care such as weight and symptoms. HeartMapp not only tracks patient performance, it provides alerts and feedback on patient performance; it also provides interventions to perform deep breathing exercises and to encourage physical activity. HeartMapp also includes HF-related education. Further exploration in a larger trial is warranted to test the feasibility of utilizing HeartMapp over time to improve short-term and long-term HF outcomes.

## Abbreviations

API: application programming interface
CHF: congestive heart failure
FSSQ: Functional Social Support Questionnaire
HF: heart failure
IMB: information-motivation-behavioral skills
iOS: iPhone operating system
IRB: institutional review board
KCCQ: Kansas City Cardiomyopathy Questionnaire
MCIR: Modified Cumulative Illness Rating scale
mHealth: mobile Health
MoCA: Montreal Cognitive Assessment
NYHA: New York Heart Association
PHQ-9: Patient Health Questionnaire
RA: research assistant
RCT: randomized controlled trial

## References

[1] Benjamin EJ, Blaha MJ, Chiuve SE, Cushman M, Das SR, Deo R, de Ferranti SD, Floyd J, Fornage M, Gillespie C, Isasi CR, Jiménez MC, Jordan LC, Judd SE, Lackland D, Lichtman JH, Lisabeth L, Liu S, Longenecker CT, Mackey RH, Matsushita K, Mozaffarian D, Mussolino ME, Nasir K, Neumar RW, Palaniappan L, Pandey DK, Thiagarajan RR, Reeves MJ, Ritchey M, Rodriguez CJ, Roth GA, Rosamond WD, Sasson C, Towfighi A, Tsao CW, Turner MB, Virani SS, Voeks JH, Willey JZ, Wilkins JT, Wu JH, Alger HM, Wong SS, Muntner P, American Heart Association Statistics Committee and Stroke Statistics Subcommittee (2017). Heart disease and stroke statistics-2017 update: a report from the American Heart Association. Circulation.

[2] Yancy CW, Jessup M, Bozkurt B, Butler J, Casey Jr DE, Drazner MH, Fonarow GC, Geraci SA, Horwich T, Januzzi JL, Johnson MR, Kasper EK, Levy WC, Masoudi FA, McBride PE, McMurray JJ, Mitchell JE, Peterson PN, Riegel B, Sam F, Stevenson LW, Tang WH, Tsai EJ, Wilkoff BL (2013). 2013 ACCF/AHA guideline for the management of heart failure: executive summary: a report of the American College of Cardiology Foundation/American Heart Association Task Force on practice guidelines. Circulation.

[3] Riegel B, Moser DK, Anker SD, Appel LJ, Dunbar SB, Grady KL, Gurvitz MZ, Havranek EP, Lee CS, Lindenfeld J, Peterson PN, Pressler SJ, Schocken DD, Whellan DJ, American Heart Association Council on Cardiovascular Nursing, American Heart Association Council on Cardiovascular Nursing, American Heart Association Council on Clinical Cardiology, American Heart Association Council on Nutrition‚ Physical Activity‚Metabolism, American Heart Association Interdisciplinary Council on Quality of CareOutcomes Research (2009). State of the science: promoting self-care in persons with heart failure: a scientific statement from the American Heart Association. Circulation.

[4] Hwang B, Moser DK, Dracup K (2014). Knowledge is insufficient for self-care among heart failure patients with psychological distress. Health Psychol.

[5] Darling C, Saczynski JS, McManus DD, Lessard D, Spencer FA, Goldberg RJ (2013). Delayed hospital presentation in acute decompensated heart failure: clinical and patient reported factors. Heart Lung.

[6] Wu JR, Frazier SK, Rayens MK, Lennie TA, Chung ML, Moser DK (2013). Medication adherence, social support, and event-free survival in patients with heart failure. Health Psychol.

[7] Fitzgerald AA, Powers JD, Ho PM, Maddox TM, Peterson PN, Allen LA, Masoudi FA, Magid DJ, Havranek EP (2011). Impact of medication nonadherence on hospitalizations and mortality in heart failure. J Card Fail.

[8] Buck HG, Lee CS, Moser DK, Albert NM, Lennie T, Bentley B, Worrall-Carter L, Riegel B (2012). Relationship between self-care and health-related quality of life in older adults with moderate to advanced heart failure. J Cardiovasc Nurs.

[9] Inglis SC, Clark RA, Dierckx R, Prieto-Merino D, Cleland J (2015). Structured telephone support or non-invasive telemonitoring for patients with heart failure. Cochrane Database Syst Rev.

[10] Koehler F, Winkler S, Schieber M, Sechtem U, Stangl K, Böhm M, Boll H, Baumann G, Honold M, Koehler K, Gelbrich G, Kirwan BA, Anker SD, Telemedical Interventional Monitoring in Heart Failure Investigators (2011). Impact of remote telemedical management on mortality and hospitalizations in ambulatory patients with chronic heart failure: the telemedical interventional monitoring in heart failure study. Circulation.

[11] Ong MK, Romano PS, Edgington S, Aronow HU, Auerbach AD, Black JT, De Marco T, Escarce JJ, Evangelista LS, Hanna B, Ganiats TG, Greenberg BH, Greenfield S, Kaplan SH, Kimchi A, Liu H, Lombardo D, Mangione CM, Sadeghi B, Sadeghi B, Sarrafzadeh M, Tong K, Fonarow GC, Better Effectiveness After Transition–Heart Failure (BEAT-HF) Research Group (2016). Effectiveness of remote patient monitoring after discharge of hospitalized patients with heart failure: the Better Effectiveness After Transition -- Heart Failure (BEAT-HF) randomized clinical trial. JAMA Intern Med.

[12] Fairbrother P, Ure J, Hanley J, McCloughan L, Denvir M, Sheikh A, McKinstry B, Telescot programme team (2014). Telemonitoring for chronic heart failure: the views of patients and healthcare professionals - a qualitative study. J Clin Nurs.

[13] Hanlon P, Daines L, Campbell C, McKinstry B, Weller D, Pinnock H (2017). Telehealth interventions to support self-management of long-term conditions: a systematic metareview of diabetes, heart failure, asthma, chronic obstructive pulmonary disease, and cancer. J Med Internet Res.

[14] Broderick A, Haque F (2015). Mobile health and patient engagement in the safety net: a survey of community health centers and clinics. Issue Brief (Commonw Fund).

[15] Carman KL, Dardess P, Maurer M, Sofaer S, Adams K, Bechtel C, Sweeney J (2013). Patient and family engagement: a framework for understanding the elements and developing interventions and policies. Health Aff (Millwood).

[16] Fisher WA, Fisher JD, Harman J, Suls J, Watson KA (2009). The information-motivation-behavioral skills model: a general social psychological approach to understanding and promoting health behavior. Social Psychological Foundations of Health and Illness.

[17] Kokonozi A, Astaras A, Semertzidis P, Michail E, Filos D, Chouvarda I, Grossenbacher O, Koller J-M, Leopoldo R, Porchet J-A, Correvon M, Luprano J, Sipilä A, Zamboulis C, Maglaveras N (2010). Development and clinical evaluation of a physiological data acquisition device for monitoring and exercise guidance of heart failure and chronic heart disease patients. http://ieeexplore.ieee.org/document/5738169/?section=abstract.

[18] Athilingam P, Labrador MA, Remo EF, Mack L, San Juan AB, Elliott AF (2016). Features and usability assessment of a patient-centered mobile application (HeartMapp) for self-management of heart failure. Appl Nurs Res.

[19] Chou R, Dana T, Bougatsos C (2009). Screening older adults for impaired visual acuity: a review of the evidence for the U.S. Preventive Services Task Force. Ann Intern Med.

[20] Bailey IL, Lovie JE (1976). New design principles for visual acuity letter charts. Am J Optom Physiol Opt.

[21] Nasreddine ZS, Phillips NA, Bédirian V, Charbonneau S, Whitehead V, Collin I, Cummings JL, Chertkow H (2005). The Montreal Cognitive Assessment, MoCA: a brief screening tool for mild cognitive impairment. J Am Geriatr Soc.

[22] Athilingam P, King KB, Burgin SW, Ackerman M, Cushman LA, Chen L (2011). Montreal Cognitive Assessment and Mini-Mental Status Examination compared as cognitive screening tools in heart failure. Heart Lung.

[23] Jordan ET, Ray EM, Johnson P, Evans WD (2011). Text4Baby: using text messaging to improve maternal and newborn health. Nurs Womens Health.

[24] Thakkar J, Kurup R, Laba TL, Santo K, Thiagalingam A, Rodgers A, Woodward M, Redfern J, Chow CK (2016). Mobile telephone text messaging for medication adherence in chronic disease: a meta-analysis. JAMA Intern Med.

[25] Lainscak M, Blue L, Clark AL, Dahlström U, Dickstein K, Ekman I, McDonagh T, McMurray JJ, Ryder M, Stewart S, Strömberg A, Jaarsma T (2011). Self-care management of heart failure: practical recommendations from the Patient Care Committee of the Heart Failure Association of the European Society of Cardiology. Eur J Heart Fail.

[26] Lam C, Smeltzer SC (2013). Patterns of symptom recognition, interpretation, and response in heart failure patients: an integrative review. J Cardiovasc Nurs.

[27] Moser DK, Lee KS, Wu JR, Mudd-Martin G, Jaarsma T, Huang TY, Fan XZ, Strömberg A, Lennie TA, Riegel B (2014). Identification of symptom clusters among patients with heart failure: an international observational study. Int J Nurs Stud.

[28] Dolgin M, Dolgin M, New York Heart Association. Criteria Committee (1994). Nomenclature and criteria for diagnosis of diseases of the heart and great vessels.

[29] Nundy S, Razi RR, Dick JJ, Smith B, Mayo A, O'Connor A, Meltzer DO (2013). A text messaging intervention to improve heart failure self-management after hospital discharge in a largely African-American population: before-after study. J Med Internet Res.

[30] Mack WJ, Freed DM, Williams BW, Henderson VW (1992). Boston Naming Test: shortened versions for use in Alzheimer's disease. J Gerontol.

[31] Balthazar ML, Cendes F, Damasceno BP (2008). Semantic error patterns on the Boston Naming Test in normal aging, amnestic mild cognitive impairment, and mild Alzheimer's disease: is there semantic disruption?. Neuropsychology.

[32] Kleiger RE, Stein PK, Bigger Jr JT (2005). Heart rate variability: measurement and clinical utility. Ann Noninvasive Electrocardiol.

[33] Billman GE (2011). Heart rate variability - a historical perspective. Front Physiol.

[34] Rustad JK, Stern TA, Hebert KA, Musselman DL (2013). Diagnosis and treatment of depression in patients with congestive heart failure: a review of the literature. Prim Care Companion CNS Disord.

[35] Jerath R, Edry JW, Barnes VA, Jerath V (2006). Physiology of long pranayamic breathing: neural respiratory elements may provide a mechanism that explains how slow deep breathing shifts the autonomic nervous system. Med Hypotheses.

[36] Cahalin LP, Mathier MA, Semigran MJ, Dec GW, DiSalvo TG (1996). The six-minute walk test predicts peak oxygen uptake and survival in patients with advanced heart failure. Chest.

[37] Hayward J, Chansin G (2016). IDTechEx.

[38] Dickson VV, Riegel B (2009). Are we teaching what patients need to know? Building skills in heart failure self-care. Heart Lung.

[39] Athilingam P, Osorio RE, Kaplan H, Oliver D, O'neachtain T, Rogal PJ (2016). Embedding patient education in mobile platform for patients with heart failure: theory-based development and beta testing. Comput Inform Nurs.

[40] Bandura A, Pajares F, Urdan T (2006). Guide for constructing self-efficacy scales. Self-efficacy beliefs of adolescents.

[41] Riegel B, Carlson B, Moser DK, Sebern M, Hicks FD, Roland V (2004). Psychometric testing of the self-care of heart failure index. J Card Fail.

[42] Morisky DE, Green LW, Levine DM (1986). Concurrent and predictive validity of a self-reported measure of medication adherence. Med Care.

[43] Reilly CM, Higgins M, Smith A, Gary RA, Robinson J, Clark PC, McCarty F, Dunbar SB (2009). Development, psychometric testing, and revision of the Atlanta Heart Failure Knowledge Test. J Cardiovasc Nurs.

[44] Green CP, Porter CB, Bresnahan DR, Spertus JA (2000). Development and evaluation of the Kansas City Cardiomyopathy Questionnaire: a new health status measure for heart failure. J Am Coll Cardiol.

[45] Pressler SJ, Subramanian U, Perkins SM, Gradus-Pizlo I, Kareken D, Kim J, Ding Y, Sauvé MJ, Sloan R (2011). Measuring depressive symptoms in heart failure: validity and reliability of the patient health questionnaire-8. Am J Crit Care.

[46] Festa JR, Jia X, Cheung K, Marchidann A, Schmidt M, Shapiro PA, Mancini DM, Naka Y, Deng M, Lantz ER, Marshall RS, Lazar RM (2011). Association of low ejection fraction with impaired verbal memory in older patients with heart failure. Arch Neurol.

[47] Athilingam P, D'Aoust RF, Miller L, Chen L (2013). Cognitive profile in persons with systolic and diastolic heart failure. Congest Heart Fail.

[48] Miller MD, Paradis CF, Houck PR, Mazumdar S, Stack JA, Rifai AH, Mulsant B, Reynolds 3rd CF (1992). Rating chronic medical illness burden in geropsychiatric practice and research: application of the Cumulative Illness Rating Scale. Psychiatry Res.

[49] Broadhead WE, Gehlbach SH, de Gruy FV, Kaplan BH (1988). The Duke-UNC Functional Social Support Questionnaire. Measurement of social support in family medicine patients. Med Care.

[50] Richardson JTE (2011). Eta squared and partial eta squared as measures of effect size in educational research. Educational Research Review.

[51] Masterson Creber RM, Maurer MS, Reading M, Hiraldo G, Hickey KT, Iribarren S (2016). Review and analysis of existing mobile phone apps to support heart failure symptom monitoring and self-care management using the Mobile Application Rating Scale (MARS). JMIR Mhealth Uhealth.

[52] Jurgens CY, Lee CS, Reitano JM, Riegel B (2013). Heart failure symptom monitoring and response training. Heart Lung.

[53] Jaarsma T, van der Wal MH, Lesman-Leegte I, Luttik ML, Hogenhuis J, Veeger NJ, Sanderman R, Hoes AW, van Gilst WH, Lok DJ, Dunselman PH, Tijssen JG, Hillege HL, van Veldhuisen DJ, Coordinating Study Evaluating Outcomes of Advising and Counseling in Heart Failure (COACH) Investigators (2008). Effect of moderate or intensive disease management program on outcome in patients with heart failure: Coordinating Study Evaluating Outcomes of Advising and Counseling in Heart Failure (COACH). Arch Intern Med.

[54] Masterson Creber R, Patey M, Lee CS, Kuan A, Jurgens C, Riegel B (2016). Motivational interviewing to improve self-care for patients with chronic heart failure: MITI-HF randomized controlled trial. Patient Educ Couns.

[55] Feltner C, Jones CD, Cené CW, Zheng ZJ, Sueta CA, Coker-Schwimmer EJ, Arvanitis M, Lohr KN, Middleton JC, Jonas DE (2014). Transitional care interventions to prevent readmissions for persons with heart failure: a systematic review and meta-analysis. Ann Intern Med.

[56] Cockayne S, Pattenden J, Worthy G, Richardson G, Lewin R (2014). Nurse facilitated self-management support for people with heart failure and their family carers (SEMAPHFOR): a randomised controlled trial. Int J Nurs Stud.

[57] Soran OZ, Piña IL, Lamas GA, Kelsey SF, Selzer F, Pilotte J, Lave JR, Feldman AM (2008). A randomized clinical trial of the clinical effects of enhanced heart failure monitoring using a computer-based telephonic monitoring system in older minorities and women. J Card Fail.

[58] Kitsiou S, Paré G, Jaana M (2015). Effects of home telemonitoring interventions on patients with chronic heart failure: an overview of systematic reviews. J Med Internet Res.

[59] Suh MK, Evangelista LS, Chen V, Hong WS, Macbeth J, Nahapetian A, Figueras FJ, Sarrafzadeh M (2010). WANDA B.: Weight and Activity with blood pressure monitoring system for heart failure patients. IEEE Trans Inf Technol Biomed.

[60] Riegel B, Dickson VV, Hoke L, McMahon JP, Reis BF, Sayers S (2006). A motivational counseling approach to improving heart failure self-care: mechanisms of effectiveness. J Cardiovasc Nurs.

[61] Singh K, Drouin K, Newmark LP, Rozenblum R, Lee J, Landman A, Pabo E, Klinger EV, Bates DW (2016). Developing a framework for evaluating the patient engagement, quality, and safety of mobile health applications. Issue Brief (Commonw Fund).

[62] Gerber T, Olazabal V, Brown K, Pablos-Mendez A (2010). An agenda for action on global e-health. Health Aff (Millwood).

[63] Gell NM, Rosenberg DE, Demiris G, LaCroix AZ, Patel KV (2015). Patterns of technology use among older adults with and without disabilities. Gerontologist.

